# Therapeutic Drug Monitoring of Certolizumab Pegol: Can Real-World Data Translate to Clinical Practice?

**DOI:** 10.1093/crocol/otab020

**Published:** 2021-05-21

**Authors:** Robert Battat

**Affiliations:** Jill Roberts Center for IBD, Division of Gastroenterology and Hepatology, New York Presbyterian Hospital/Weill-Cornell Medical College, New York, New York, USA

Certolizumab pegol is a humanized, tumor necrosis factor (TNF) antagonist that is an effective therapy for moderate to severe Crohn disease (CD).^1–4^ Utilization of therapeutic drug monitoring, in which TNF antagonists drug concentrations and antidrug antibodies are measured, has been shown to improve treatment outcomes in inflammatory bowel disease. Certolizumab differs from other TNF antagonist due to it being free of the Fc region and conjugated to polyethylene glycol. Additionally, compared to other TNF antagonists, pharmacokinetic data on certolizumab are more limited. Thus, there has been a need to increase available data on the relationship between certolizumab pharmacokinetics and patient outcomes.

In this issue of *Crohn’s & Colitis 360*, Ramos et al describe a real-world, single-center experience on the association of certolizumab drug and antidrug antibody concentrations with outcomes in CD.^5^ This retrospective study increases available real-world data on certolizumab exposure–response relationships during maintenance therapy.

The authors outline that the mean certolizumab concentration was 18.9 µg/mL. Additionally, 27.3% of patients had positive antidrug antibodies and these were associated with significantly lower drug concentrations. Higher certolizumab concentrations were associated with clinical response (symptom-based) and radiologic response. A threshold of 18.9 µg/mL was also the optimal threshold to differentiate clinical response. Median certolizumab concentrations were higher in those achieving clinical (30.4 vs 10.3 µg/mL, *P* = 0.0015) and radiologic response (29.6 vs 5.8 µg/mL; *P* = 0.006) compared to nonresponders.

Certolizumab concentrations above 18.9 µg/mL were associated with an odds ratio of 5.5 of achieving clinical response, whereas antidrug antibodies were associated with reduced odds for this outcome (odds ratio = 0.20). In a subgroup of patients with available data, numerically higher certolizumab concentrations existed in those with mucosal healing, deep remission, and radiographic healing (27.8–29.6 µg/mL) compared to those not achieving these outcomes (10.3–12.0 µg/mL). Although these did not achieve statistical significance, the similar numerical differences between all outcomes suggest sample size may have affected the latter analyses.

Previously, Vande Casteele et al analyzed population pharmacokinetic models based on pooled data from 9 clinical trials of certolizumab in CD to simulate drug concentrations in over 2000 patients. Week 12 certolizumab concentration of 14.8 µg/mL was associated with subsequently achieving both CD activity index <150 and fecal calprotectin <250 g/g at week 26.^6^ The proportion of patients with remission increased from 58% to 74% using certolizumab thresholds from ≥10 to ≥20 µg/mL, respectively.^7^

Pharmacokinetic data from a single-arm, open-label study in CD patients (MUSIC trial) demonstrated that higher week 8 certolizumab concentrations were associated with favorable outcomes at week 10. These included endoscopic response (*P* = 0.0016; area under the receiver operating curve = 0.70) and clinical remission (*P* = 0.0302, area under the receiver operating curve = 0.69). The highest certolizumab concentration quartile (>23.3 µg/mL) had higher rates of clinical and endoscopic remission and no plateau was observed.^8^ Data were lacking on maintenance serum certolizumab concentrations due to the limited sample size (n = 18) of patients with available data during at this time point.

In both the previous population pharmacokinetic study and the MUSIC trial, limited data existed on the relationship between outcomes and serum concentrations measured during the maintenance phase. Additionally, radiographic data were lacking. Thus, the present study adds data on the exposure–response relationships in this phase of therapy in addition to analyzing for a novel outcome.

Interestingly, the current study found similar thresholds (18.9 µg/mL) for favorable outcomes to population pharmacokinetic studies (20 µg/mL) and the guidelines on which they are based.^6,7^ Symptom-based clinical outcomes are often discordant from objective endoscopic outcomes and endoscopy better predicts important outcomes such as hospitalization and surgery. Thus, it is noteworthy that data were limited on the relationship between maintenance certolizumab and endoscopic outcomes. Future studies with more robust sample sizes are needed to delineate if the measurement of maintenance certolizumab drug concentrations are associated with improved rates of mucosal healing, deep remission, and radiographic healing. While the present study utilized absence of ulcers, an important endoscopic treatment outcome, future studies are required using validated clinical, endoscopic, and radiographic outcomes such as the CD-patient reported outcome (CD PRO)-2, the simplified endoscopic score for CD (SES-CD), and the simplified magnetic resonance index of activity (MARIA) score for CD.^9,10^ Lastly, transmural healing is an emerging endpoint that still requires further study prior to routine incorporation into treat-to-target algorithms.^11^

The authors should be congratulated for these real-world data to assist in moving toward the utilization of maintenance certolizumab drug concentrations to guide clinical practice. Similar certolizumab concentration thresholds were found to be associated with outcomes compared to previous studies, and this study provides data on measurement of maintenance concentrations. Nonetheless, adequately powered studies are still needed to better understand the relationship between maintenance certolizumab concentrations with outcomes using validated indices for endpoint assessment.

## Notes

Funding: Robert Battat declares research support from the fund for the Future award and Jill Roberts Funds at the Department of Medicine, Weill Cornell Medicine.
